# Probe-based qPCR as an alternative to modified Knott’s test when screening dogs for heartworm (*Dirofilaria immitis*) infection in combination with antigen detection tests

**DOI:** 10.1186/s13071-022-05372-x

**Published:** 2022-08-29

**Authors:** Veronica Negron, Meriam N. Saleh, Caroline Sobotyk, Joe L. Luksovsky, Tatiani V. Harvey, Guilherme G. Verocai

**Affiliations:** Department of Veterinary Pathobiology, College of Veterinary Medicine and Biomedical Sciences, Texas A&M University, 4467 TAMU, College Station, TX 77843-4467 USA

**Keywords:** Antigen testing, Canine filariosis, *Dirofilaria immitis*, Heartworm disease, Microfilariae detection test, Molecular diagnostics, qPCR

## Abstract

**Background:**

Current recommendations for diagnosis of *Dirofilaria immitis* infection in dogs rely on the detection of antigen produced largely by adult females coupled with the visualization of microfilariae (mf) in the circulation via a microfilaria detection test (MFDT). It is hypothesized that qPCR assays used in parallel with antigen detection tests will perform better in detecting mf than modified Knott’s test (MK), when combined with antigen detection. This study compares probe-based qPCR and MK techniques for mf detection used in parallel with the DiroCHEK^®^ antigen test to screen for heartworm infection in shelter dogs.

**Methods:**

Matching blood and serum samples were collected from 300 shelter dogs in Brazos and Harris counties, Texas, USA. Blood was assessed for the presence of mf via MK and the presence of *D. immitis* DNA by a species-specific probe-based qPCR assay. Serum samples were tested for the presence of heartworm antigen using DiroCHEK^®^ before and after immune complex dissociation (ICD) via heat treatment. In addition, the performance of each diagnostic test was evaluated via Chi-square test, Cochran’s *Q* test, and post hoc analysis.

**Results:**

Qualitatively, MK detected mf in 22.0% (66/300) of samples, 55 of which were morphologically identified as *D. immitis* and 11 as *Acanthocheilonema reconditum*. The range of heartworm mf was 28 to 88,803 mf/ml (median: 6627.5). Real-time PCR detected *D. immitis* DNA in 20.7% (62/300) of samples. Heartworm antigen was detected in 24.7% (74/300) of samples pre-ICD, and in 29.3% (88/300) post-ICD. When comparing tests, the Chi-square and McNemar’s tests showed that the difference between positive and negative proportions was statistically significant. The Cochran test showed the difference in the distributions of cases and non-cases was significant when individual tests were combined (*χ*^2^ = 62.3, *df* = 3, *P* < 0.0001) and when parallel methods were combined (*χ*^2^ = 43.1, *df* = 4, *P* < 0.0001).

**Conclusion:**

Considering individual and combined test performances, practicality, and efficient use of bench time, this heartworm-specific probe-based qPCR method is a viable option as a mf detection test to be used in parallel with antigen tests for canine heartworm infection in diagnostic and research settings.

**Graphical Abstract:**

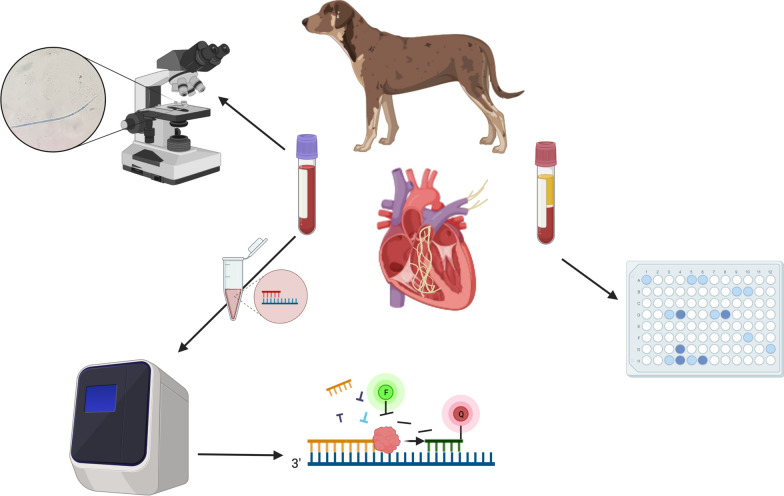

## Background

*Dirofilaria immitis* is a zoonotic filarioid nematode with a worldwide distribution and considered endemic or hyper-endemic in many countries within the Americas, Europe, and Asia [1, 2]. In dogs, *D. immitis* infection can result in clinical disease, including caval syndrome, hemolysis, liver and kidney dysfunction, heart failure, and death [3]. Due to the severity of canine heartworm disease and the increased prevalence rate over past decades, surveys have focused on the diagnosis and prevalence of *D. immitis* in dogs and wild canids that may serve as a reservoir host [4–6]. Despite the higher risk of complications and the increased risk of infection in endemic areas, canine heartworm disease is preventable. Accurate and efficient diagnostic screening is a crucial step for successful canine heartworm prevention and remains a challenge for veterinary clinicians and practitioners, especially in areas considered co-endemic for *D. immitis* and other filarial nematodes.

Diagnosis of *D. immitis* infection in dogs relies on the detection of circulating antigen that is largely produced by adult female worms coupled with a microfilaria detection test (MFDT). The American Heartworm Society (AHS) and the Companion Animal Parasite Council (CAPC) both currently recommend this combination of diagnostic tests when screening dogs for heartworm infection [3, 7]. There are a variety of MFDT in use with varying sensitivities, including the direct smear, microhematocrit tube test or buffy coat examination, and the modified Knott’s (MK) test. The direct smear and microhematocrit tube test are less sensitive testing methods and do not allow for species-level identification of microfilaria (mf) present [8], but are more often used in practice settings. The MK test is a more sensitive method for detecting mf as it is a concentration technique and allows for assessment of mf morphology under microscopy, which is necessary to differentiate *D. immitis* from other filarioid nematodes [8, 9]. Diagnostic laboratories routinely utilize MK to distinguish mf species based on size and morphology [10]. However, the accuracy of morphologically identifying mf is dependent on the skill of the observer [9]. Additional clinical diagnostic tests could also help veterinarians to confirm heartworm infection and understand the disease outcome and patient’s prognosis, including radiography and echocardiography [3].

Better diagnostic methods are needed to detect and accurately confirm the presence of *D. immitis* microfilaremia. This will remove observer subjectivity and therefore improve the detection and diagnosis of canine heartworm infections. The aim of this study was to compare the performance of a probe-based quantitative polymerase chain reaction (qPCR) for *D. immitis* DNA to the MK technique in detecting heartworm mf when used in conjunction with the DiroCHEK^®^ antigen test. An additional goal was to determine whether the qPCR could be used in place of MK as a more sensitive, and potentially more efficient, test in diagnostic and research laboratories.

## Methods

### Population and sample collection

Matching whole blood and serum samples were collected from 300 shelter dogs between February and August 2020 in Brazos and Harris counties of Texas, (USA). Inclusion criteria consisted of no recent history of macrocyclic lactone administration and an estimated age of greater than 6 months. Serum samples were stored at –20 °C until antigen testing, and EDTA blood samples were stored at 2 °C until MK was performed.

### Modified Knott’s Test (MK)

Microfilariae were quantified and morphologically identified using the MK test as described by Rojas et al. [9] and Zajac et al. [8] with the following modifications. Briefly, 0.5 ml of EDTA blood was added to 4.5 ml of 2% formalin solution in a 15 ml test tube then homogenized using a vortex mixer to ensure lysis of red blood cells. Tubes containing the mixture were centrifuged at 1600 relative centrifugal force (RCF) for 5 min. After centrifugation, 4.0 ml of supernatant was removed, taking care to not disrupt the pellet at the bottom of the tube. The sediment was mixed with 35 µl of 0.1% methylene blue to stain any mf. An aliquot of 20 µl was placed on a glass slide and covered with a 22 × 22 mm coverslip. The entire coverslip was visually scanned, and mf were quantified on low power (100×), if present. Morphology was assessed at high power (400×) for species identification. In the absence of mf in the 20 µl aliquot, the remaining sediment was scanned in 200 µl portions with a 40 × 22 mm coverslip. If the entire sediment was read, total mf were counted, and the total was adjusted for blood volume to be measured in total microfilariae per milliliter (mf/ml). If mf were found in the 20 µl aliquot, total mf/µl was calculated with the following formula as described by Rojas et al. [9]:

$$mf/\mu L= \frac{mf \ observed \times \left\{\left[\left( {V}_{blood}+{V}_{formalin}\right)-{V}_{supernatant}\right]+{V}_{methylene\ blue}\right\}+{V}_{methylene\ blue}\}}{{V}_{sample} \times {V}_{blood}}$$

### Antigen detection test pre- and post-ICD

Serum samples were tested for *D. immitis* antigen using a commercial, qualitative enzyme-linked immunosorbent assay (ELISA) test, DiroCHEK^®^ (Zoetis Inc., Kalamazoo, MI, USA). All serum samples were antigen tested pre-immune complex dissociation (ICD), and samples with sufficient volume were also antigen tested post-ICD via heat treatment. Antigen testing and interpretation were performed according to the manufacturer’s instructions to assess samples for visual color change to indicate antigen detection. Wells were considered positive for the detection of *D. immitis* antigen if a color change was visible after 5 min of incubation. Wells with no color change were considered no antigen detected (NAD). For the ICD, heat treatment was performed as described by Swartzentruber et al. [11]. Briefly, 250 µl of serum was heated at 104 °C for 10 min in a dry heat block and centrifuged for 10 min at 16,000 RCF. The resulting supernatant was used as template for post-ICD antigen detection via DiroCHEK^®^ as described above.

### DNA extraction and probe-based qPCR

Genomic DNA was extracted and purified from 200 µl EDTA whole blood using the QIAmp DNA Blood Mini Kit (QIAGEN, Hilden, Germany), following the manufacturer’s instructions. All samples were screened for the presence of *D. immitis* DNA using a probe-based qPCR assay targeting a fragment of the cytochrome c oxidase subunit 1 (*cox1*) of the mitochondrial DNA, with primers and probe previously described by Laidoudi et al. [12] and protocol modified by Sobotyk et al. [4]. A 166-base-pair *cox1* fragment was amplified using the forward primer Fil.COI.749-F (5′-CAT CCT GAG GTT TAT GTT ATT ATT TT-3′) and reverse Fil.COI.914-R (5′-CWG TAT ACA TAT GAT GRC CYC A-3′), and TaqMan^®^ probe D.imm.COI.777-P (6FAM-CGG TGT TTG GGA TTG TTA GTG-MGB) designed by Laidoudi et al. [12]. All reactions were performed in a total volume of 20 μl containing 5 μl of genomic DNA template, 10 μl (2×) of TaqMan^®^ Fast Advanced Master Mix (Applied Biosystems, Waltham, MA, USA), 50 μM of each primer, and 20 μM of probe.

Real-time PCR reactions were performed on a QuantStudio 3 real-time PCR system (Applied Biosystems, Waltham, MA, USA) using the using TaqMan^®^ universal cycling conditions. Cycling conditions consisted of a hold stage at 95 °C for 20 s and 40 cycles of a two-step PCR stage at 95 °C for 1 s followed by 60 °C for 20 s [4]. All runs included a positive and a non-template control. The positive control consisted of DNA extracted from an adult *D. immitis*. The non-template control consisted of nuclease-free water.

### Statistical analysis

The data was analyzed by multiple methods. The absolute and relative frequencies of positive samples were calculated for each diagnostic method and for the relevant parallel combination of diagnostic methods utilizing Epi Info™ 7.2.5. The difference between paired proportions was compared using the paired McNemar’s test and either Chi-square (*χ*^*2*^) statistic or Fisher’s exact test. In the Chi-square test, residues outside the range −2.73 to 2.73 were considered significant, and the residue limit was corrected according to MacDonald and Gardner [13]. The 95% confidence interval for rates was calculated as described previously [14–16]. The agreement between tests was calculated by Cohen’s kappa coefficient (κ) [17, 18]. To estimate agreement between two diagnostic tests for these cases, prevalence- and bias-adjusted κ (PABAK) that expresses κ in terms of bias index (BI), prevalence index (PI) and observed agreement (*p*o) was calculated. BI, PI, and po were also calculated. BI is an index of the bias between tests, and PI, an index of the differences between the overall proportion of positive and negative outputs. The higher the absolute value of either PI or BI, the higher the influence of bias and prevalence on the κ value. [19, 20]. Combinations of tests were performed in parallel. In this case, positive dogs by test 1 or 2 or both were considered cases, while non-cases were those negative by both tests. Cochran’s *Q* test was used to verify whether the discrepancies between the individually paired or combined tests were significant, and post hoc analysis was performed if the *P*-value was less than 0.05. The *P-*values were adjusted according to MacDonald and Gardner [13]. Bonferroni correction was applied to adjust the *P*-values. These analyses were performed in R Studio version 1.2.5033.

## Results

Of the 300 dogs screened, 29.7% (89/300) tested positive for *D. immitis* infection on at least one diagnostic test (Table 1). Overall, DiroCHEK^®^ detected the greatest number of cases, followed by qPCR and MK test. Qualitatively, MK detected mf in 22.0% (66/300) of samples, of which 55 (18.3%; 55/300) were morphologically identified as *D. immitis* and 11 (3.7%; 11/300) as *Acanthocheilonema reconditum*. The heartworm microfilaremia determined by MK test ranged from 28 to 88,803 mf/ml (median: 6727.5 mf/ml). In comparison, the probe-based qPCR found seven additional heartworm-positive samples, detecting *D. immitis* DNA in 20.7% (62/300) of the whole blood samples (Table 2). Heartworm antigen was detected by DiroCHEK^®^ in 24.7% (74/300) and 29.3% (88/300) of serum samples pre- and post-ICD, respectively. Of the 74 antigen-positive dogs without heat treatment of serum, only one become negative after ICD.

**Table 1:** Diagnostic test results for *D. immitis*-positive dogs (*n* = 89) from three different shelters in Texas, USA

No. of samples	MK	qPCR	DiroCHEK^®^ pre-ICD	DiroCHEK^®^ post-ICD
54	+	+	+	+
1	+	+	−	+
5	−	** + **	** + **	** + **
2	−	+	−	+
14	−	−	+	+
1	−	−	+	−
12	−	−	−	+

*MK* modified Knott’s test

*qPCR* real-time PCR

*ICD* immune complex dissociation

**Table 2:** Frequency of *D. immitis*-positive cases by individual and parallel diagnostic methods (*n* = 300)

Diagnostic method(s)	*D. immitis*-positive dogs (*n*)	Frequency % (95% CI)
MK	55	18.3 (14.4–23.1)
qPCR	62	20.7 (16.5–25.6)
DiroCHEK^®^ pre-ICD	74	24.7 (20.1–29.8)
DiroCHEK^®^ post-ICD	88	29.3 (24.5–34.7)
MK + DiroCHEK^®^ pre-ICD	75	25.9 (20.4–30.2)
MK + DiroCHEK^®^ post-ICD	88	29.3 (24.5–34.7)
qPCR + DiroCHEK^®^ pre-ICD	77	25.7 (21.0–30.9)
qPCR + DiroCHEK^®^ post-ICD	88	29.3 (24.5–34.7)

*MK* modified Knott’s test; *qPCR* real-time PCR; *ICD* immune complex dissociation

As shown in Table 2, the association of MFDT and DiroCHEK^®^ detected higher numbers of *D. immitis*-positive dogs when compared with MK, qPCR, and DiroCHEK^®^ pre-ICD methods alone. Real-time PCR when associated with DiroCHEK^®^ pre-ICD detected 25.7% (77/300) of heartworm cases, which is slightly higher than the MK test and DiroCHEK^®^ pre-ICD combination (25.0%, 75/300). Performing DiroCHEK^®^ post-ICD alone or associated with MK or qPCR presented the highest number of *D. immitis* detections (29.3%, 88/300).

The Chi-square test showed that the proportions of cases and non-cases between the methods (Table 3), individually, were different and statistically significant. However, the analysis of adjusted standardized residues identified that such differences (*χ*^2^ = 11.2, *df* = 3, *P < *0.004) are basically due to the higher frequency of cases detected by the DiroCHEK^®^ post-ICD and a lower frequency of cases detected by MK.

**Table 3:** Comparison between tests for the detection of *D. immitis* in shelter dogs (*n* = 300) from Texas, USA

Compared tests (Test 1/Test 2)	+/+ (a)	+/− (b)	−/+ (c)	−/− (d)	*k*	95% CI *k*	*p*_o_	BI	PI	PABAK	∆ (%)	*P*-value
MK/qPCR*	55	0	7	238	0.92	0.87–0.98	0.97	−0.02	0.61	0.95	2.34	0.023**
MK/DiroCHEK^®^ pre-ICD *	54	1	20	225	0.79	0.71–0.88	0.93	−0.06	0.57	0.86	6.34	< 0.001**
MK/DiroCHEK^®^ post-ICD *	55	0	33	212	0.70	0.61–0.79	0.89	−0.11	0.52	0.78	11	< 0.001**
qPCR/DiroCHEK^®^ pre-ICD *	59	3	15	223	0.83	0.75–0.90	0.94	−0.04	0.55	0.88	4	< 0.001**
qPCR/DiroCHEK^®^ post-ICD *	62	0	26	212	0.77	0.69–0.85	0.91	−0.09	0.50	0.82	8.66	< 0.001**
DiroCHEK^®^ pre-ICD/ DiroCHEK^®^ post-ICD *	73	1	15	211	0.86	0.80–0.93	0.95	−0.04	0.46	0.89	4.66	0.0012**

*MK* modified Knott’s test; *ICD* immune complex dissociation; *qPCR* real-time PCR; (+) positive result; (−) negative result; CI confidence interval; po observed agreement [po = (a + d)/n]; BI Byrt’s bias index [BI = (b–c)/n]; PI Byrt’s prevalence asymmetry index [PI = (a–d)/n]; PABAK prevalence- and bias-adjusted kappa; Δ difference in paired proportions, calculated using McNemar’s test

* *P*-value < 0.05 (Chi-square test)

** statistically significant (McNemar's test)

McNemar’s test (Table 3) and Cochran’s test, in the pairing of individual (*χ*^2^ = 62.3, *df* = 3, *P < *0.0001) and combined methods (*χ*^2^ = 43.1, *df* = 4, *P < *0.0001), showed that the discrepancies between some tests were significant and were also significant after Bonferroni correction (Table 4). Increased cases on DiroCHEK^®^ post-ICD contributed to the most significant discrepancies in the parallel paired combinations (Table 4).

**Table 4:** Cochran's *Q* test and pairwise McNemar's test for individual and parallel diagnostic methods in the investigation of *Dirofilaria immitis* in Texas shelter dogs (*n* = 300)

Method combinations		*P*-value
Comparison of individual methods	*χ*^2^ = 62.3, *df* = 3	< 0.0001
MK		
qPCR		
DiroCHEK^®^ pre-ICD		
DiroCHEK^®^ post-ICD		
Post hoc analysis		
MK/qPCR		0.008
DiroCHEK^®^ pre-ICD/ DiroCHEK^®^ post-ICD		< 0.0001*
Comparison of combined methods	*χ*^2^ = 43.1, *df* = 4	< 0.0001
MK + DiroCHEK^®^ pre-ICD(A)		
MK + DiroCHEK^®^ post-ICD (B)		
qPCR + DiroCHEK^®^ pre-ICD (C)		
qPCR + DiroCHEK^®^ post-ICD (D)		
Post hoc analysis		
A/B		< 0.0001**
A/C		0.157
B/D		1
C/D		0.002**

*MK* modified Knott’s test; *ICD* immune complex dissociation; *qPCR* real-time PCR

* Significant after Bonferroni correction (α = 0.008)

** Significant after Bonferroni correction (α = 0.005)

## Discussion

In the present study, *D. immitis* infection was detected in 29.7% of shelter dogs. The overall heartworm prevalence found in this study was higher than previous reports in owned dogs (3.1%) [7] and shelter dogs (16.0%) [21] across Texas. Several factors could be associated with this difference, including population of dogs tested, lack or lower frequency of heartworm prophylaxis, and diagnostic testing decision. In the canine population, MK is considered the preferred and most sensitive concentration method to detected and differentiate mf of *D. immitis* from other filarioid nematodes [3, 8]. However, MFDT cannot detect amicrofilaremic infections and should always be performed in association with heartworm antigen tests to prevent false negative results [3, 8]. Although the sensitivity and specificity rate vary among of the commercial heartworm antigen test kits, antigen testing is considered the most sensitive diagnostic method [3]. The ELISA methods have shown higher sensitivity and specificity rates ranging from 85.7% to 100% depending on the number of mature female *D*. *immitis* present [3, 10].

The use of molecular approaches for diagnosing heartworm infections has increased over the past years, including conventional PCR and qPCR targeting nuclear and mitochondrial genes [4, 22, 23]. The integration of molecular assays in the diagnosis of filarial infections has been shown to be an efficient and reliable screening tool to confirm the disease in intermediate and definitive hosts [23, 24]. In this study, the probe-based PCR assay detected *D. immitis* DNA in 20.7% (62/300) of samples, which was more than the 18.3% (55/300) detected by MK. Despite the lack of a statistical difference between these two tests, with the higher numbers of positive *D. immitis* detected by qPCR, it would be more informative for clinicians, as it also matched more often with the antigen test results both pre- and post-ICD. Furthermore, when both probe-based qPCR and MK were associated with heartworm antigen post-ICD, no differences in the number of positive results were found (29.3%; 88/300).

The application of this probe-based qPCR assay in combination with *D. immitis* antigen test seems to be an efficient alternative for primary diagnostic heartworm screening. The AHS recommends annual testing using both heartworm antigen test for detection of adult worms and a MFDT for detection of circulating *D. immitis* mf [3]. When combined with the antigen test without heat treatment, qPCR test identified 25.7% (77/300) of dogs as infected with heartworm in the present study, while MK detected fewer positive cases, down to 25.0% (75/300). These results demonstrate the reliability of the probe-based qPCR protocol as a routine MFDT and highlight its efficiency in detecting canine heartworm infections when associated with the heartworm antigen test.

Lane et al. [25] and Hays et al. [26] compared multiple diagnostic methods to maximize heartworm detection in dog and cat populations. In both studies, the combination of antigen and MFDT increased the proportion of positive results, but additional analysis was needed for species identification, namely conventional PCR, followed by Sanger sequencing. The use of a heartworm-specific probe eliminates the need for additional analysis for unequivocal species identification, and the specificity of the probe removes any potential error in inaccurately identifying mf species that could occur with the MK technique. As demonstrated by Laidoudi et al. [12], we also showed the species-specific detection of DNA of *D. immitis* mf DNA, and no amplification was seen on qPCR in any of the samples that tested *A. reconditum*-positive on MK. Additionally, when the probe was tested with *D. repens* DNA, no amplification was seen [12], indicating that there is no cross-reactivity across filarial species.

In the present study, 4.7% (14/300) of mf-negative samples were considered positive by DiroCHEK^®^ pre- and post-ICD. DiroCHEK^®^ post-ICD alone revealed 4.0% (12/300) positive samples that were considered mf-negative and NAD before heat treatment. The increase in positive samples post-ICD is well documented in the literature and corroborates previously published studies reporting that antigen detection post-ICD increases the number of heartworm cases detected among companion animals [10]. More specifically, ICD is generally recommended for dogs in endemic areas with no or unknown history of prevention [10], which was the case for the studied canine population. Negative *D. immitis* mf tests in dogs that tested positive in antigen detection assays have been associated with occult heartworm infections, including low or absent mf concentration during the prepatent period, sterility of female adult worms due to senility or drug-induced factors, and host immune response [27].

Previous studies have demonstrated the cross-reactivity potential of several commercial heartworm ELISA tests, before and after ICD methods, with other nematodes, including *D*. *repens*, *Angiostrongylus vasorum*, and *Spirocerca lupi* [28–30]. Only a single sample converted from positive to NAD post-ICD; this dog also tested positive to *A. reconditum* by the MK test, and negative in the probe-based qPCR. Although testing positive via antigen detection made this a heartworm-positive sample, necropsy confirmation of the *D. immitis* infection status was not performed in this study. Previous studies have also reported few cases of positive antigen results only before ICD method, indicating that heat treatment may damage or denature *D. immitis* proteins and lead to false NAD results [31, 32]. Therefore, ICD protocols should not be performed as routine heartworm screening, but only in suspected cases of heartworm disease and in specific circumstances, as mentioned in the paragraph above [3, 10].

Another factor to be considered when evaluating and implementing diagnostic tests in reference laboratories and clinical and research settings is its practicability. The probe-based qPCR assay presented here is a more efficient and less time-consuming process when compared to the MK test. In addition, the probe-based qPCR allows for high-throughput testing, with the size of the well plate used being the limiting factor for how many reactions can be run simultaneously, and requires less active bench time. In fact, the protocol and reagents used allowed the acquisition of results for an entire 96-well plate in 27 min, which is faster than the original protocol [12]. Clinically, the use of probe-based qPCR as a routine diagnostic tool could provide additional benefits for veterinarians and clients, including more convenient and faster mf detection testing and reduced time to obtain a reliable and accurate result using as low as 250 µl of whole blood. Our analysis was limited to shelter dog population with no or unknown history of macrocyclic lactone administration. However, it is possible that animals received prior veterinary care and heartworm preventive medication which could interfere with the accuracy of heartworm detection. Moreover, the *D*. *immitis* infection status of the dog population was not confirmed by necropsy in the present study. Therefore, additional studies with necropsy diagnoses are needed to establish the accuracy, specificity, and sensitivity rates of probe-based qPCR assay.

## Conclusion

The results of this study demonstrated that qPCR is a reliable and highly specific test for detection of *D. immitis* microfilaremia. When combined with DiroCHEK^®^, qPCR and MK results had comparable results, thus showing that probe-based qPCR associated with heartworm antigen testing is a viable option as a primary diagnostic screening for *D. immitis* infection in canine populations. Compared with the MK, probe-based qPCR detected a higher number of positive dogs and proved to be specific for *D. immitis* DNA and had no cross-reactivity with *A. reconditum*.

## Data Availability

All data generated or analyzed during this study are included in this published article and its supplementary information files.
